# Systems approaches to global and national physical activity plans

**DOI:** 10.2471/BLT.18.220533

**Published:** 2018-12-19

**Authors:** Harry Rutter, Nick Cavill, Adrian Bauman, Fiona Bull

**Affiliations:** a Department of Social and Policy Sciences, University of Bath, Claverton Down, Bath BA2 7AY, England.; b Centre for Exercise, Nutrition and Health Sciences, University of Bristol, Bristol, England.; c School of Public Health, Sydney University, Sydney, Australia.; d Prevention of Noncommunicable Diseases Department, World Health Organization, Geneva, Switzerland.

A key driver for promoting physical activity is reducing the global burden of noncommunicable diseases, particularly cardiovascular disease, cancer and diabetes. These diseases are responsible for more than 41 million deaths annually, of which a third occur before the age of 70 years.^1^ Physical activity has multiple positive impacts on noncommunicable diseases such as heart disease, stroke, diabetes, and breast and colon cancer,^2^ as well as numerous social and economic benefits including reduced use of fossil fuels, cleaner air and less congested, safer roads. All these effects are closely linked to several sustainable development goals.^3^ However, policy actions have been insufficient and uneven, and government strategies to increase physical activity have not consistently increased the proportion of the adult population meeting recommended levels of activity.^4^ Without significant scaling of efforts at local, regional, national and international levels, the global targets for physical activity are unlikely to be achieved.

In response to this lack of progress, there has been a growing recognition of the role of systems theory and accompanying tools such as systems mapping in helping to frame responses to complex public health challenges.^5^^–^^8^ A complex systems model of public health conceptualizes poor health and health inequalities as outcomes of a multitude of interdependent elements within a connected whole. These elements affect each other in sometimes subtle ways, with changes potentially reverberating throughout the system.^5^ In public health, systems theory has been used most extensively in work on obesity^9^^,^^10^ and is being applied to the evaluation of the soft drink industry levy in the United Kingdom of Great Britain and Northern Ireland.^11^

Systems thinking provides a framework to help examine the factors involved in a problem, the relations between these factors and changes over time; it views actions as integrated across political, social, cultural, economic and scientific domains within a system. A system is more than the sum of its parts, encompassing the interactions between these parts and the actors involved. This approach differs from traditional linear models of cause and effect that underpin much of the existing evidence base and takes account of factors such as adaptation, the ways in which a system responds to interventions within it, and feedback, which drives some of those responses.

System mapping provides a visual depiction of how the different parts of a system relate to one another. One well known example of a system map comes from the 2007 United Kingdom Government Foresight report *Tackling Obesities*, where the complex dynamic influences driving the obesity epidemic were comprehensively mapped for the first time.^9^ Similar approaches have been used for issues such as dietary inequalities^8^ and tobacco control.^12^

Physical activity promotion in recent years has increasingly adopted socioecological approaches that place the drivers of physical activity in their social and environmental context. A systems approach builds on this contextualization by adding the dynamic connections between the factors that collectively form the system, and considering the ways in which actors interact with them. A systems approach can help make sense of what otherwise might be perceived as diverse and chaotic relations between large numbers of factors and their physical, commercial, sociocultural and political contexts.

There are several potential uses for systems maps, which can provide a nuanced depiction of the multisectoral and complex nature of a problem. Paradoxically, mapping out and exposing a system by disaggregating factors that have previously been conflated, and illustrating how they interact (or proposing potential mechanisms by which they might), may enhance and simplify understanding of the elements and processes involved. In addition, maps may be used as the basis of systems dynamics and other models to explore causal mechanisms and potential impacts of interventions. Furthermore, mapping can also support the identification of data sources for monitoring and/or evaluation.

The process of collaborating to build a map can contribute to building consensus on the nature of a problem and engagement with potential policy responses required to address it. Bringing together stakeholders involved in tackling a problem can help those actors to identify their part in a system and to appreciate better the roles of others. The process of generating a system map and the insights gained by stakeholders who do so, may be more important than the map itself, which may not have wider generalizability to other contexts.

Maps may also support the identification of important opportunities to exert influence within a system. Different kinds of leverage points for influencing a system have been suggested,^13^^,^^14^ including: (i) structural factors, such as the presence of walking infrastructure; (ii) feedback mechanisms, such as the social benefits from volunteer-led community-based physical activity programmes such as Parkrun; (iii) system structures, for example the existence of a national coordinating agency for physical activity; (iv) goals, such as a national sports policy that has a stated aim of promoting physical activity across the population, beyond competitive sport; and (v) the overarching paradigms that define a system, for example treating transport policy as a tool for promoting healthy mobility, beyond the usual core focus of moving people and goods.

**Fig. 1:**
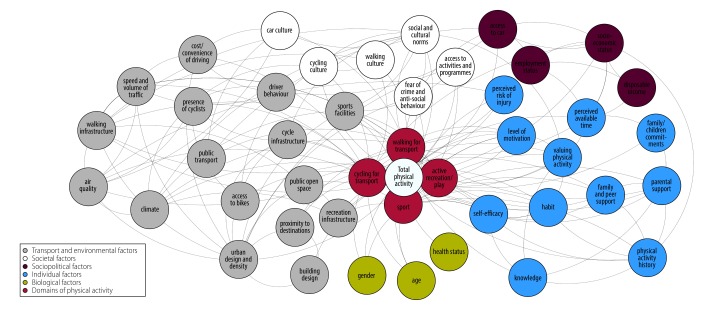
An initial physical activity system map

Note: This map provides an illustration of some of the main drivers of physical activity and inactivity, and the relations between them, based on evidence from systematic reviews and expert opinion. The map is a tool to illustrate the breadth of factors driving physical activity, and does not purport to provide a definitive description of all possible ways of conceptualizing the physical activity system.

One example of the use of such a map is seen in the development of a systems framework to support the World Health Organization Global Action Plan on Physical Activity (Fig. 1).^3^ The key determinants or correlates of physical activity behaviour from the literature were mapped^15^^–^^17^ and the draft map was reviewed by a panel of experts representing academia, civil society, medical and allied health sectors, transport and urban design, education and sports sectors and United Nations agencies, and adapted in response to their feedback.

The resulting map provides a visual representation of the multiple factors underpinning physical activity. The map is intended to be used to: support the implementation of the global plan on physical activity through identification of potential mechanisms for influencing the determinants of physical activity; support the identification of data sources for monitoring and evaluation; promote an integrated approach to physical activity policy that emphasizes the cross-sectoral relations involved; and act as the basis of visual tools for communicating the need for wide-ranging actions across multiple sectors and domains to support the promotion of physical activity. The map does not aim to be a formal causal loop diagram with balancing and reinforcing loops, nor does it attempt to quantify the nature of the relations between factors.

An integrated systems map, based on best available scientific evidence, can capture and illustrate the complex nature of the multiple factors that promote or hinder an outcome such as physical activity. Conceptual models can advance our understanding of the complexity of planning comprehensive and integrated approaches to a public health issue such as physical activity. Conceptual models can also guide both selection and prioritization of actions, and help to coordinate responses to problems.

There are limitations to this kind of tool. These maps are not generally intended to provide robust quantitative descriptions of the nature and magnitude of causal relations; rather, they set out to illustrate the multiple components of a complex system in ways that have relevance for policy-makers and practitioners. There is no definitive standard against which such a map can be assessed, and another group producing a map of the physical activity system might produce different results. To date little empirical evidence exists on the value of these maps, but research to evaluate the impacts of this kind of approach is underway. 

A systems map can support the development of policy and action plans to increase physical activity in several ways. Such maps can contribute to communicating the multiple factors and cross-sectoral nature of the influences on physical activity for policy-makers. The maps can illustrate the range of opportunities to implement policy actions across multiple areas to influence the system; demonstrate the breadth of partnerships needed (including outside the health sector); identify key areas for action that may represent opportunities for significant impacts on policy; support analysis and identification of key areas and priorities for action; support the development of tailored local-level maps that include important contextual factors; help audit existing policy actions or plan new ones; and inform monitoring and evaluation.

A key value of the map is to illustrate the multiple components of an effective response to address physical inactivity in populations. Most importantly, the map shows that the notion of a single approach to increasing physical activity is misguided and inappropriate.

System maps can extend beyond socioecological models and communicate not only the actions required for effective promotion of physical activity, but also the relations between these actions. Emphasizing the interconnectedness of the key drivers of physical inactivity explicitly demonstrates the roles that multiple sectors need to play in our collective response to noncommunicable diseases.
